# Effect of Hydrothermal and Vapor Thermal Treatments on Apatite Inductivity of Titanate Nanotubes on Anodized Ti–5Nb–5Mo Surface

**DOI:** 10.3390/nano13081296

**Published:** 2023-04-07

**Authors:** Kuan-Hsiang Hsieh, Hsueh-Chuan Hsu, Shih-Ching Wu, Yi-Cheng Shih, Hsiang-Wei Yang, Wen-Fu Ho

**Affiliations:** 1 Department of Surgery, Division of Orthopaedics, Zuoying Branch of Kaohsiung Armed Forces General Hospital, Kaohsiung 81342, Taiwan; bedbedegg2013@gmail.com; 2 Department of Dental Technology and Materials Science, Central Taiwan University of Science and Technology, Taichung 40601, Taiwan; hchsu@ctust.edu.tw (H.-C.H.); scwu@ctust.edu.tw (S.-C.W.); 3 Department of Chemical and Materials Engineering, National University of Kaohsiung, Kaohsiung 81148, Taiwan

**Keywords:** titanium alloy, anodization, hydrothermal, vapor thermal, apatite induction

## Abstract

Although titanium (Ti) alloys have been widely employed as biomedical materials, they cannot achieve satisfactory osseointegration when implanted in the human body due to their biologically inert nature. Surface modification can enhance both their bioactivity and corrosion resistance. The present study employed a Ti–5Nb–5Mo alloy with a metastable α″ phase. This alloy may undergo phase changes after conventional high-temperature heat treatment, which can deteriorate its properties. This study heat-treated the anodized Ti–5Nb–5Mo alloy by using a low-temperature hydrothermal or vapor thermal method to analyze the effects of heat treatment on its apatite induction. The results revealed that the porous nanotube structure on the surface of the alloy was transformed into anatase nanoparticles after hydrothermal or vapor thermal treatment at 150 °C for 6 h. After immersion in simulated body fluid (SBF) for 7 days, the amount of apatite deposited on the surface of the vapor thermal-treated alloy exceeded that on the hydrothermal-treated alloy. Therefore, post-heat treatment of anodized Ti–5Nb–5Mo by using the vapor thermal method can enhance its apatite inductivity without altering its structure.

## 1. Introduction

Ti–6Al–4V ELI is the most common clinical titanium (Ti) alloy implant, with its elastic modulus (114 GPa) [1] being much higher than that of the cortical bone (20–40 GPa) [2]. If the elastic modulus of an implant material exceeds that of the human bone, it can cause a stress shielding effect, resulting in bone loss and the loosening of the implant [3,4]. Therefore, researchers have developed several new Ti alloys with low elastic moduli in the past few decades. Ti–Nb-based alloys have attracted much research attention due to their lower elastic moduli and enhanced biocompatibility; furthermore, they exhibit shape memory behavior and superelasticity [5]. In recent years, our research group has developed a Ti–5Nb–5Mo alloy with an α″ phase. This alloy has a lower modulus of elasticity (62 GPa), and its bending strength to elastic modulus ratio is as high as 24.7, which is higher than those of commercially pure Ti (8.5) and the developed Ti–5Nb alloy (15.4) by 191% and 60%, respectively [6]. In this study, Mo was primarily added given its nontoxicity, non-allergenicity, and strong β-stabilizing effect [6,7,8].

A crucial obstacle is associated with the use of Ti alloys in implant applications. Ti alloys are bioinert and do not bond well to bones after implantation into the human body [9]. In recent years, researchers have adopted multiple surface modification methods, including electrospinning [10], sol–gel [11], sputtering [12], electrophoretic deposition [13], plasma electrolytic oxidation (PEO) [14,15,16], and anodic oxidation [17] methods. Among them, the anodization method can produce ordered TiO_2_ nanotubes, which have a large specific surface area [18]. Additionally, nanotubes provide sites with superior apatite-forming ability and cell activity, which facilitates bone tissue growth and enhances bone formation [19]. Generally, amorphous TiO_2_ on anodized Ti alloys is transformed into the anatase or rutile phase through subsequent thermal annealing [20]. Rutile and anatase are the most widely used phases in many fields of applications. Rutile is the most thermodynamically stable form, while anatase is the low temperature form. Moreover, heat treatment can increase the hydrophilicity of the surface of Ti alloys, which can effectively enhance their bioactivity [21]. However, excessively high-temperature heat treatment causes phase changes of the Ti–5Nb–5Mo alloy with a metastable α″ phase, which should be avoided because such phase changes can cause its mechanical properties to deteriorate [22]. The crystallization temperature of the anatase phase is lower than that of the rutile phase. Additionally, the anatase phase has a more compatible lattice match with hydroxyapatite (HA); therefore, it facilitates the nucleation and growth of apatite more effectively than the rutile phase [23].

Yu et al. [24] calcined TiO_2_ nanotube array films by using vapor thermal and hydrothermal methods. Their results indicated that the vapor thermal-treated sample maintained its nanotubular morphology with higher crystallinity and larger crystal size; therefore, that sample exhibited the highest photocatalytic activity. However, few studies have examined the apatite induction of nanotubes treated with the vapor thermal method. The present study subjected an anodized Ti–5Nb–5Mo sample to post-heat treatment by using hydrothermal or vapor thermal methods to investigate its morphology, microstructure, and apatite induction. Through low-temperature heat treatment, the nanotubes on the surface of the alloy can not only develop a crystalline film, but it can avoid the phase change of the metastable α″ phase and the deterioration of the mechanical properties of the alloy. Additionally, this study is the first to investigate the apatite induction on the surface of the nanotubes after hydrothermal or vapor thermal treatment. Therefore, this investigation exhibits scientific and practical significance for the development of surface modification technology for biomedical Ti alloys.

## 2. Materials and Methods

This study prepared a Ti–5Nb–5Mo alloy (wt %) by using arc melting and a casting system (A-028, DAWNSHINE, Taoyuan, Taiwan) with an Ar atmosphere. The alloy was composed of Ti (99.7% pure), Nb (99.95% pure), and Mo (99.95% pure) in accordance with their weight percentages. All the metals were purchased from Ultimate Materials Technology Co., Ltd., Taiwan. The weight of each ingot was approximately 15 g, and the alloy sample was obtained by casting after repeated smelting for five times. The size of the specimen after cutting was 15 × 15 × 1 mm^3^. The sample was first ground to #1200 with sandpaper and then chemically etched with 4 wt % HF (Union Chemical, Hsinchu, Taiwan) + 5 M HNO_3_ (Showa, Tokyo, Japan) solution for 30 s. Next, the sample was cleaned using ethanol three times and deionized water twice alternately in an ultra-sonic bath for 10 min each time. Finally, the sample was dried at 45 °C for 2 h.

A three-electrode potentiostat (BP-4002, Beam, Taichung, Taiwan) was employed for anodization, with platinum as the auxiliary electrode, the Ti alloy sample as the working electrode, and a saturated calomel electrode as the reference electrode. The anodic potential was set at 10 V, and 0.15 M NH_4_F (Carlo Erba, Val de Reuil, France) was used as the electrolyte under magnetic stirring for 90 min at room temperature. After anodization, the sample was placed in deionized water, washed in an ultra-sonic bath (DC200H, DELTA, New Taipei City, Taiwan) for 10 min, and finally dried at 45 °C for 2 h.

This study placed the anodized sample in a Teflon bottle for post-treatment by using the hydrothermal or vapor thermal method. For the hydrothermal method, the anodized sample was placed in a Teflon bottle containing deionized water; after locking the Teflon lid, the sample was placed in an autoclave for hydrothermal treatment. For vapor thermal treatment, the anodized sample was placed in a Teflon bottle without deionized water with the lid unlocked; the bottle was then placed in an autoclave filled with deionized water up to 80% of its capacity. The experimental conditions of the hydrothermal and vapor thermal reactions are listed in Table 1.

The surface morphology of the specimens after hydrothermal or vapor thermal treatment were observed through scanning electron microscopy (SEM; S-3000N, Hitachi, Japan). Element analysis was conducted through energy-dispersive X-ray spectroscopy (EDS) in SEM. Phase analysis was completed using X-ray diffraction (XRD; MXP-III, Brukers, Leipzig, Germany); the operating voltage was fixed at 40 V, the operating current was fixed at 40 mA, and low grazing-angle diffraction was employed.

The hydrophilicity of the sample surface was measured using a goniometer (model 100SB, Sindatek, Taipei, Taiwan) with automated pipetting and imaging systems (1 μL deionized water). Three specimens were used for each experimental condition, and the average value was then calculated. Statistical significance was set at * *p* < 0.05, ** *p* < 0.005 and assessed using one-way analysis of variance and Tukey’s test for multiple comparisons.

The aforementioned hydrothermal- or vapor thermal-treated samples were soaked in simulated body fluid (SBF) to evaluate their apatite induction. This study used SBF in accordance with the ion concentrations proposed by Kokubo and Takadama [25], which resembles that in the human plasma; the pH value was adjusted to 7.4 by using 1 M HCl (Showa, Tokyo, Japan). The SBF was prepared by dissolving reagent grade NaCl (Daejung, Siheung, Korea), NaHCO_3_ (Showa, Tokyo, Japan), KCl (Showa, Tokyo, Japan), K_2_HPO_4_·3H_2_O (Acros Organics, Geel, Belgium), MgCl_2_·6H_2_O (Showa, Tokyo, Japan), CaCl_2_ (Showa, Tokyo, Japan), and Na_2_SO_4_ (Showa, Tokyo, Japan) into distilled water. The samples were immersed in 50 mL of SBF for 2 and 7 days, respectively, at 37.5 °C in a water tank. The solution was changed every 2 days to maintain the ion concentrations of the SBF. After soaking for the specified durations, the samples were rinsed with deionized water for 30 s and finally dried at 45 °C for 2 days. The surfaces of the samples were analyzed using XRD, SEM, and EDS after soaking in SBF to evaluate their apatite inductivity.

## 3. Results and Discussion

**Figure 1:**
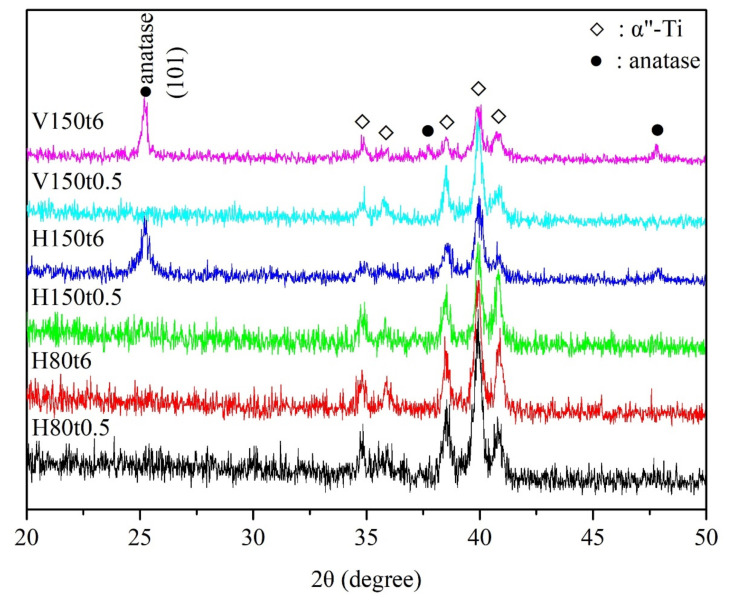
XRD patterns of anodized Ti–5Nb–5Mo under various hydrothermal or vapor thermal treatments.

The XRD patterns of anodized Ti–5Nb–5Mo after hydrothermal or vapor thermal treatment under various conditions are illustrated in Figure 1. The sample that was hydrothermal-treated at 80 °C for 0.5 and 6 h (H80t0.5 and H80t6) had no crystalline phase, except for the α” phase of the alloy substrate, indicating that the titania nanotubes on the surface of the sample remained amorphous. When hydrothermal or vapor thermal treatment was conducted at 150 °C for 0.5 h (H150t0.5 and V150t0.5), titania diffraction peaks of the nanotubes were not observed. However, when hydrothermal or vapor thermal treatment was conducted at 150 °C for 6 h (H150t6 and V150t6), distinct diffraction peaks were detected in the anatase phase, which indicated the crystalline phase of titania nanotubes. This study employed the full width at half maximum intensity of the (101) diffraction peak of anatase to calculate the crystallite size [26]; the results revealed that the crystallite sizes of H150t6 and V150t6 were 25.38 nm and 25.28 nm, respectively. Additionally, the crystallinity of V150t6 was 1.21 times greater than that of H150t6 when the (101) diffraction peak was used to calculate the relative crystallinity of the titania nanotubes [27]. Fischer et al. [27] reported that the crystallite size and crystallinity of anatase increase as the hydrothermal or vapor thermal time increase. Furthermore, as the crystallinity of titania increases, the apatite-forming ability of the Ti sample soaked in SBF also increases [28,29]. Therefore, the apatite inductivity of the vapor thermal-treated sample is superior to that of the hydrothermal-treated sample.

**Figure 2:**
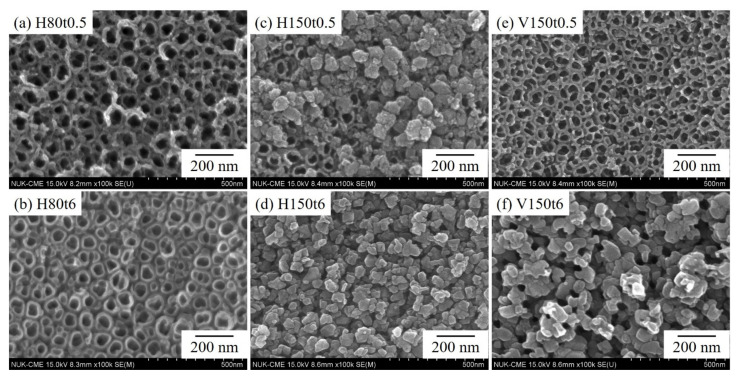
SEM images of anodized Ti–5Nb–5Mo under various hydrothermal or vapor thermal treatments: (**a**) H80t0.5, (**b**) H80t6, (**c**) H150t0.5, (**d**) H150t6, (**e**) V150t0.5, and (**f**) V150t6.

The SEM photographs of the Ti–5Nb–5Mo sample after anodization and hydrothermal or vapor thermal treatment under various conditions are presented in Figure 2. After hydrothermal treatment at 80 °C for 0.5 h (H80t0.5), the nanotube morphology remained intact, with no obvious precipitate on its surface (Figure 2a). After 6 h of treatment (H80t6), precipitates were observed along the orifice edges of the nanotubes (Figure 2b). Moreover, the observed nanotube structure was not continuous but rather had a discrete structure with gaps between adjacent nanotubes and a thickened tube wall. After the hydrothermal reaction conducted at 150 °C for 0.5 h (H150t0.5), some granular precipitates with a diameter of approximately 49 μm covered the underlying nanotubes, but nanotubes were still observed in some areas (Figure 2c). After hydrothermal treatment for 6 h (H150t6), the nanotube surface was completely covered by anatase titania particles with a diameter of approximately 54 μm (Figure 2d). After the sample was annealed through vapor thermal treatment at 150 °C for 0.5 h (V150t0.5), the surface of the sample exhibited still-intact nanotube morphology with a continuous wall between the nanotubes (Figure 2e), resembling the morphology of H80t0.5 (Figure 2a). After 6 h of vapor thermal treatment (V150t6), many granular anatase titania particles with a diameter of approximately 58 μm appeared on the surface; however, many small poles could also be observed on the surface (Figure 2f).

Some studies have revealed that amorphous titania nanotubes begin to transform to the anatase phase above 300 °C through calcination in air [30,31,32]. In the present study, small anatase nanoparticles appeared on the tops and walls of the nanotubes at 150 °C due to hydrothermal or vapor thermal reactions; they then gradually expanded to cover the entire surface of the nanotubes (Figure 2d,f). Low-temperature hydrothermal and vapor thermal treatments are more efficient methods for the preparation of highly crystallized thin titania films [24]. Because the total Gibbs free energy of a titania particle includes the free energy of its bulk and surface, the nucleation process is primarily dominated by surface Gibbs free energy [33]. Thus, the phase transformation to anatase nanoparticles occurs due to the nucleation and nuclei growth of anatase crystallites. Yu et al. [21] hypothesized that the presence of water in the reaction system catalyzes the crystallization process and strongly influences the morphology of amorphous titania nanotubes.

**Figure 3:**
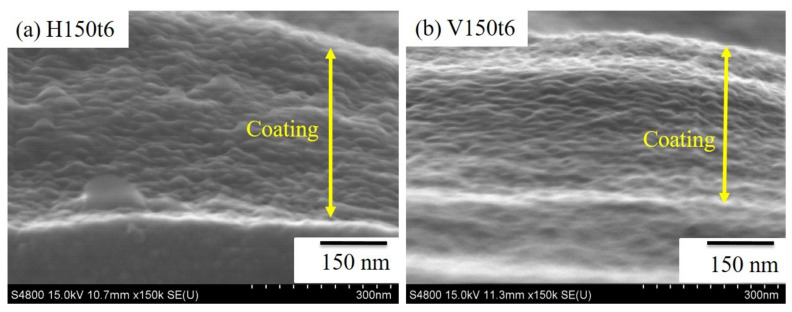
Cross-sectional SEM images of anodized Ti–5Nb–5Mo under various hydrothermal or vapor thermal treatments: (**a**) H150t6 and (**b**) V150t6.

Figure 3 is cross-sectional SEM photos of H150t6 and V150t6. It can be observed that after hydrothermal and vapor thermal treatment, both the samples presented granular titania particles, and the morphology of nanotubes obtained by anodic oxidation treatment had completely disappeared.

**Figure 4:**
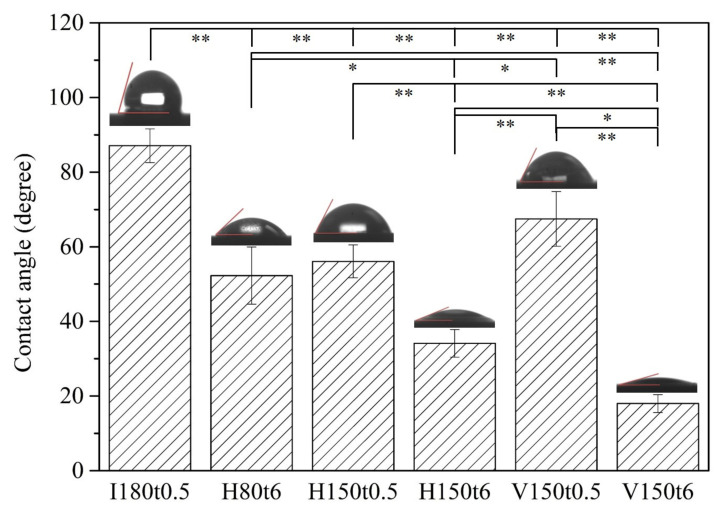
Water contact angles of anodized Ti–5Nb–5Mo under various hydrothermal or vapor thermal treatments. Statistical significance (*) was set at a *p*-value of < 0.05, and highly significant (**) as *p* < 0.005.

The contact angles of water droplets on anodized samples after hydrothermal or vapor thermal treatment under various conditions are illustrated in Figure 4. The results revealed that the contact angle decreased as the heating time increased, regardless of the method. The samples undergoing hydrothermal or vapor thermal treatment at 150 °C for 6 h (H150t6 and V150t6) had low contact angles of 34.1° and 18.0°, respectively. Because the surface area of Ti alloys increases after anodization, it provides a greater area for the absorption and interaction of water molecules. Enhanced hydrophilicity after heat treatment primarily results from an increase in the crystallinity of titania and changes in surface morphology. Heat treatment promotes the migration of oxygen and oxygen vacancy sites in the titania lattice, and many vacancy sites are generated on the titania surface. These vacancy sites are conducive to the absorption of OH groups, thereby increasing hydrophilicity [34]. In addition, the crystallinity of nanotubular titania affects the hydrophilicity of the annealed surfaces [35]. When a Ti alloy possesses a greater hydrophilic surface, its surface absorbs protein more easily, which greatly enhances its apatite induction. Fibroblasts exhibit better attachment and spreading on hydrophilic surfaces than on hydrophobic surfaces [36].

**Figure 5:**
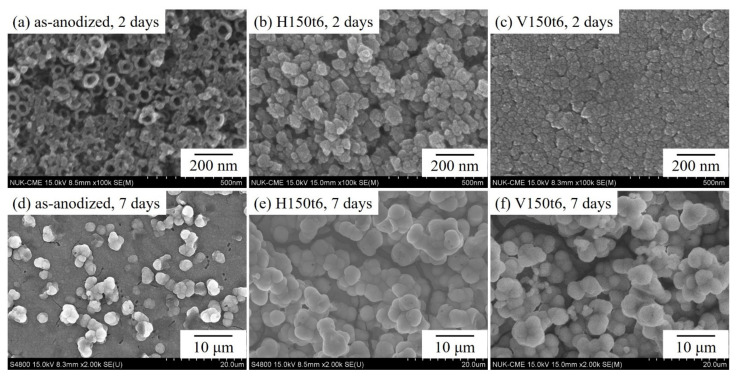
SEM images of anodized Ti–5Nb–5Mo under hydrothermal or vapor thermal treatments at 150 °C for 6 h after soaking in SBF for 2 or 7 days: (**a**) as-anodized, 2 days, (**b**) H150t6, 2 days, (**c**) V150t6, 2 days, (**d**) as-anodized, 7 days, (**e**) H150t6, 7 days, and (**f**) V150t6, 7 days.

Figure 5 presents the SEM photographs of anodized specimens (as-anodized) that underwent hydrothermal or vapor thermal treatment at 150 °C for 6 h (H150t6 and V150t6) and were then immersed in SBF for 2 or 7 days. The as-anodized samples (Figure 5a) constituted the control group. Inspection of Figure 4a reveals that the as-anodized sample had only a small amount of apatite precipitates, and the nanotube remained clearly observable on the sample surface. However, after immersion in SBF for 2 days, a large amount of apatite formed on the surfaces of hydrothermal-treated (Figure 5b) and vapor thermal-treated (Figure 5c) specimens, indicating that the post-treated alloy possessed excellent apatite induction. After soaking in SBF for 7 days, the as-anodized sample (Figure 5d) was completely covered with apatite. However, the apatite particles on the surface of the samples (Figure 5e,f), treated with the hydrothermal or vapor thermal method, grew even larger. Consequently, after hydrothermal or vapor thermal treatment, nanotubular titanate exhibited superior apatite induction to that of the as-anodized sample. Xiao et al. [37] compared the apatite induction of titania nanotubes with and without hydrothermal treatment in a saturated Ca(OH)_2_ solution. Their results indicated that anatase titanate exhibited superior apatite inductivity because it can be hydrolyzed to produce more Ti-OH in SBF, which enhances apatite precipitation. Furthermore, the amount of apatite deposited on hydrothermal-treated or vapor thermal-treated specimens cannot be clearly distinguished using SEM images; therefore, EDS and XRD were employed to analyze the surfaces of the specimens soaked in SBF.

**Figure 6:**
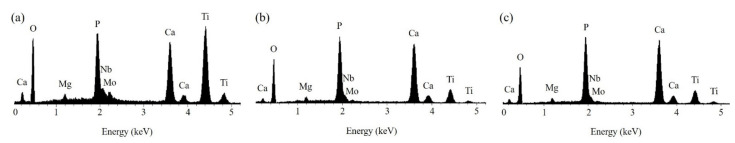
EDS results (mapping mode) of anodized Ti–5Nb–5Mo under hydrothermal or vapor thermal treatments at 150 °C for 6 h after soaking in SBF for 7 days: (**a**) as-anodized, (**b**) H150t6, and (**c**) V150t6.

Figure 6 presents the SEM/EDS mapping mode of the anodized Ti–5Nb–5Mo alloy (as-anodized), which underwent hydrothermal or vapor thermal treatment at 150 °C for 6 h (H150t6 and V150t6) after soaking in SBF for 7 days. The results revealed greatly elevated intensities of Ca and P on the surfaces of the samples after hydrothermal or vapor thermal treatment, indicating a greater amount of apatite deposits. Relatively lower intensities of Ca and P and a greater intensity of Ti were detected in the as-anodized sample, which indicated that less apatite was formed. Additionally, the hydrothermal- or vapor thermal-treated samples exhibited a low-intensity Ti peak, indicating that the thick apatite layer weakened the signal of the X-ray emission. According to the SEM/EDS results, the Ca/P molar ratios of the three sample types (as-anodized, H150t6, and V150t6) were 1.48, 1.62, and 1.66, respectively. A Ca/P molar ratio of apatite close to 1.67 (stoichiometric HA) indicates good apatite inductivity on the surface of the Ti sample [38]. After immersion in SBF for 7 days, the surfaces of both H150t6 and V150t6 were completely covered by apatite (Figure 5e,f), and their Ca/P molar ratios were close to 1.67, indicating enhanced apatite induction, particularly in the vapor thermal-treated sample (V150t6).

**Figure 7:**
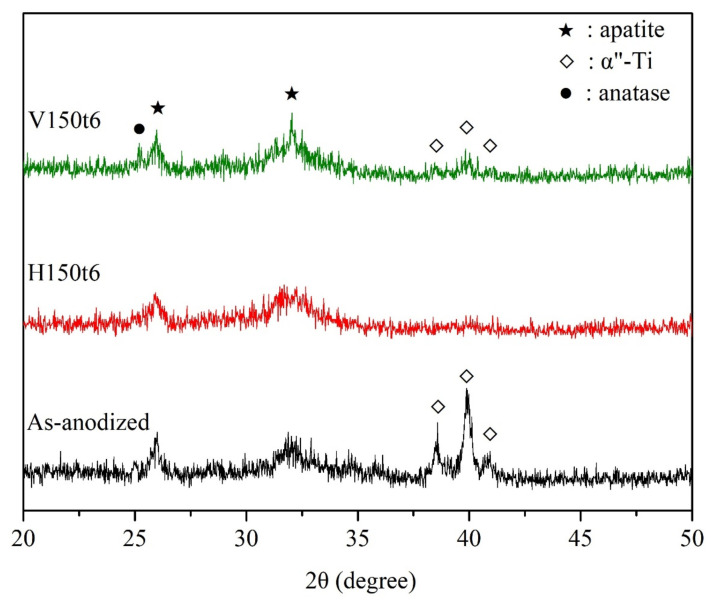
XRD patterns of anodized Ti–5Nb–5Mo under hydrothermal or vapor thermal treatments at 150 °C for 6 h after soaking in SBF for 7 days.

Figure 7 shows the XRD patterns of the anodized samples (as-anodized) that underwent hydrothermal or vapor thermal treatment at 150 °C for 6 h (H150t6 and V150t6) after soaking in SBF for 7 days. The results demonstrated that the peak intensities of the α”-Ti phase for the as-anodized sample were much higher than those of the other two heat-treated samples (H150t6 and V150t6). The decrease in the diffraction peak intensities of the α”-Ti phase for H150t6 and V150t6 can be attributed to the thicker apatite layers that formed on their surfaces. Additionally, the surface of the vapor thermal-treated sample (V150t6) had a sharp diffraction peak of apatite (31.76°), indicating its high crystallinity. This result is consistent with the EDS results, which revealed that V150t6 had a greater Ca/P molar ratio (1.66) than the other sample types. The present study results indicated that amorphous nanotubular titanate can be transformed into the anatase crystalline phase through hydrothermal or vapor thermal treatment; furthermore, the surface of the anodized Ti–5Nb–5Mo alloy exhibited favorable apatite-forming ability. Among the study sample types, the vapor thermal-treated sample exhibited superior apatite inductivity.

After hydrothermal or vapor thermal treatment, the nanotubes on the surface of the Ti–5Nb–5Mo alloy were hydrated to form significantly high amount of Ti–OH groups. Ti–OH groups can absorb Ca^2+^, OH^−^, PO_4_^3−^, and HPO_4_^2−^ ions in SBF through electrostatic bonding, and promote the deposition of apatite. For all types of heat treatment of Ti alloys, the mechanism of apatite formation on the surface is the same. However, Tsuchiya et al. [39] indicated that crystal structure and surface morphology are more effective factors than OH groups for the formation of apatite. Therefore, V150t6 had a better apatite inductivity in this study, probably due to the higher crystallinity of the anatase (Figure 1).

## 4. Conclusions

This study prepared nanotubular titanate on the surface of a Ti–5Nb–5Mo alloy with low elastic moduli through anodization and subsequent hydrothermal or vapor thermal treatment. Additionally, the apatite induction of the thermal-annealed samples was evaluated after soaking in SBF. The results revealed amorphous nanotubes on the surface of as-anodized samples, and significant diffraction peaks of anatase titania were observed after hydrothermal or vapor thermal treatment at 150 °C for 6 h (H150t6 and V150t6). Moreover, the anatase phase of the V150t6 sample had higher crystallinity than that of the H150t6 sample; the water contact angle test also demonstrated that the V150t6 sample had superior hydrophilicity to the H150t6 sample. After immersion in SBF for 7 days, apatite deposits formed on the surfaces of all specimens, but the SEM, EDS, and XRD results indicated that the V150t6 sample exhibited the best apatite-forming ability. Therefore, both hydrothermal and vapor thermal treatment (H150t6 and V150t6) enhanced the apatite induction of nanotubular titanate on the surface of the Ti–5Nb–5Mo alloy after anodization, but vapor thermal treatment yielded superior apatite inductivity.

## Figures and Tables

**Table 1:** Sample code and experimental conditions under hydrothermal or vapor thermal treatments.

Sample Code	Hydrothermal	Vapor Thermal
H80t0.5	80 °C, 0.5 h	
H80t6	80 °C, 6 h	
H150t0.5	150 °C, 0.5 h	
H150t6	150 °C, 6 h	
V150t0.5		150 °C, 0.5 h
V150t6		150 °C, 6 h

## Data Availability

Not applicable.

## References

[1] Bittredge O., Hassanin H., El-Sayed M.A., Eldessouky H.M., Alsaleh N.A., Alrasheedi N.H., Essa K., Ahmadein M. (2022). Fabrication and Optimisation of Ti-6Al-4V Lattice-Structured Total Shoulder Implants Using Laser Additive Manufacturing. Materials.

[2] Wang P., Todai M., Nakano T. (2019). Beta titanium single crystal with bone-like elastic modulus and large crystallographic elastic anisotropy. Mater. Sci. Eng. A.

[3] Fouda N., Mostafa R., Saker A. (2019). Numerical study of stress shielding reduction at fractured bone using metallic and composite bone-plate models. Ain Shams Eng. J..

[4] Luo C., Liu Y., Peng B., Chen M., Liu Z., Li Z., Kuang H., Gong B., Li Z., Sun H. (2023). PEEK for Oral Applications: Recent Advances in Mechanical and Adhesive Properties. Polymers.

[5] Pilz S., Hariharan A., Günther F., Zimmermann M., Gebert A. (2023). Influence of isothermal omega precipitation aging on deformation mechanisms and mechanical properties of a β-type Ti-Nb alloy. J. Alloys Compd..

[6] Hsu H.C., Wu S.C., Hsu S.K., Kao W.H., Ho W.F. (2013). Structure and mechanical properties of as-cast Ti–5Nb-based alloy with Mo addition. Mater. Sci. Eng. A.

[7] Zhou Y.L., Luo D.M. (2011). Corrosion behavior of Ti–Mo alloys cold rolled and heat treated. J. Alloys Compd..

[8] Wong K.K., Hsu H.C., Wu S.C., Ho W.F. (2021). Structure and properties of Ti-rich Ti-Zr-Nb-Mo medium-entropy alloys. J. Alloys Compd..

[9] Vishwakarma V., Kaliaraj G.S., Amirtharaj Mosas K.K. (2023). Multifunctional Coatings on Implant Materials—A Systematic Review of the Current Scenario. Coatings.

[10] Şimşek M., Aldemir S.D., Gümüşderelioğlu M. (2019). Anticellular PEO coatings on titanium surfaces by sequential electrospinning and crosslinking processes. Emerg. Mater..

[11] Shanmugapriya, Sivamaran V., Padma Rao A., Senthil Kumar P., Selvamani S.T., Mandal T.K. (2022). Sol–gel derived Al2O3/Gr/HAP nanocomposite coatings on Ti–6Al–4V alloy for enhancing tribo-mech properties and antibacterial activity for bone implants. Appl. Phys. A.

[12] Huang H.L., Tsai M.T., Chang Y.Y., Lin Y.J., Hsu J.T. (2020). Fabrication of a novel Ta(Zn)O thin film on titanium by magnetron sputtering and plasma electrolytic oxidation for cell biocompatibilities and antibacterial applications. Metals.

[13] Kumari R., Yadav K.B., Barole S., Archana K., Besra L.D. (2022). Microstructural characterisation and wettability behaviour of nano-HA coating on Ti-6Al-4V alloy by electrophoretic deposition method (EPD). Adv. Mater. Process. Technol..

[14] Mashtalyar D.V., Nadaraia K.V., Plekhova N.G., Imshinetskiy I.M., Piatkova M.A., Pleshkova A.I., Kislova S.E., Sinebryukhov S.L., Gnedenkov S.V. (2022). Antibacterial Ca/P-coatings formed on Mg alloy using plasma electrolytic oxidation and antibiotic impregnation. Mater. Lett..

[15] Imshinetskiy I., Kashepa V., Nadaraia K., Mashtalyar D., Suchkov S., Zadorozhny P., Ustinov A., Sinebryukhov S., Gnedenkov S. (2023). PEO Coatings Modified with Halloysite Nanotubes: Composition, Properties, and Release Performance. Int. J. Mol. Sci..

[16] Kaseem M., Choe H.C. (2023). Synchronized Improvements in the Protective and Bioactive Properties of Plasma-Electrolyzed Layers via Cellulose Microcrystalline. ACS Biomater. Sci. Eng..

[17] Ocampo R.A., Echeverria F.E. (2019). Effect of the anodization parameters on TiO_2_ nanotubes characteristics produced in aqueous electrolytes with CMC. Appl. Surf. Sci..

[18] Pishkar N., Ghoranneviss M., Ghorannevis Z., Akbari H. (2018). Study of the highly ordered TiO_2_ nanotubes physical properties prepared with two-step anodization. Results Phys..

[19] Khrunyk Y.Y., Belikov S.V., Tsurkan M.V., Vyalykh I.V., Markaryan A.Y., Karabanalov M.S., Popov A.A., Wysokowski M. (2020). Surface-Dependent Osteoblasts Response to TiO_2_ Nanotubes of Different Crystallinity. Nanomaterials.

[20] Savitha R., Raghunathan R., Chetty R. (2023). Enhanced visible light sensitized photoreaction by mixed phase titania nanotubes. Appl. Surf. Sci..

[21] Kim S.Y., Jin G.C., Kim Y.K., Kao W.H., Park I.S. (2014). Effect of alkali and heat treatments for bioactivity of TiO_2_ nanotubes. Appl. Surf. Sci..

[22] Shimagami K., Ito T., Toda Y., Yumoto A., Yamabe-Mitarai Y. (2019). Effects of Zr and Si addition on high-temperature mechanical properties and microstructure in Ti–10Al–2Nb-based alloys. Mat. Sci. Eng. A.

[23] Kim S.P., Kaseem M., Choe H.C. (2020). Plasma electrolytic oxidation of Ti–25Nb–xTa alloys in solution containing Ca and P ions. Surf. Coat. Technol..

[24] Yu J., Dai G., Cheng B. (2010). Effect of crystallization methods on morphology and photocatalytic activity of anodized TiO_2_ nanotube array films. J. Phys. Chem. C.

[25] Kokubo T., Takadama H. (2006). How useful is SBF in predicting in vivo bone bioactivity?. Biomaterials.

[26] Yu J., Wang B. (2010). Effect of calcination temperature on morphology and photoelectrochemical properties of anodized titanium dioxide nanotube arrays. Appl. Catal. B.

[27] Fischer K., Gläser R., Schulze A. (2014). Nanoneedle and nanotubular titanium dioxide–PES mixed matrix membrane for photocatalysis. Appl. Catal. B.

[28] Yang B., Uchida M., Kim H.M., Zhang X., Kokubo T. (2004). Preparation of bioactive titanium metal via anodic oxidation treatment. Biomaterials.

[29] Wei D., Qing W.F., Zhou R., Li B., Wang Y., Zhou Y., Jia D. (2015). Titania nanotube/nano-brushite composited bioactive coating with micro/nanotopography on titanium formed by anodic oxidation and hydrothermal treatment. Ceram. Int..

[30] Getz M.N., Chatzitakis A., Liu X., Carvalho P.A., Bjorheim T.S., Norby T. (2020). Voids in walls of mesoporous TiO_2_ anatase nanotubes by controlled formation and annihilation of protonated titanium vacancies. Mater. Chem. Phys..

[31] Aeimbhu A. (2018). Effect of calcination temperature on morphology, wettability and anatase/rutile phase ratio of titanium dioxide nanotube arrays. Mater. Today Proc..

[32] Suwannaruang T., Kamonsuangkasem K., Kidkhunthod P., Chirawatkul P., Saiyasombat C., Chanlek N., Wantala K. (2018). Influence of nitrogen content levels on structural properties and photocatalytic activities of nanorice-like N-doped TiO_2_ with various calcination temperatures. Mater. Res. Bull..

[33] Ding K.L., Miao Z.J., Hu B.J., An G.M., Sun Z.Y., Han B.X., Liu Z.M. (2010). Study on the anatase to rutile phase transformation and controlled synthesis of rutile nanocrystals with the assistance of ionic liquid. Langmuir.

[34] Lima G.G., Souza G.B., Lepienski C.M., Kuromoto N.K. (2016). Mechanical properties of anodic titanium films containing ions of Ca and P submitted to heat and hydrothermal treatment. J. Mech. Behav. Biomed. Mater..

[35] Roguska A., Pisarek M., Belcarz A., Marconc L., Holdynski M., Andrzejczuk M., Janik-Czachora M. (2016). Improvement of the bio-functional properties of TiO_2_ nanotubes. Appl. Sur. Sci..

[36] Ponsonnet L., Reybier K., Jaffrezic N., Comte V., Lagneau C., Lissac M., Martelet C. (2003). Relationship between surface properties (roughness, wettability) of titanium and titanium alloys and cell behaviour. Mater. Sci. Eng. C.

[37] Xiao X., Liang J., Tang H., Yang X., Liu R., Chen Y. (2013). Preparation and bioactivity of TiO_2_ nanotube arrays containing calcium and phosphorus. Appl. Surf. Sci..

[38] Burant B., Robak J., Leniart A., Piwonski I., Batory D. (2017). The effect of concentration and source of calcium ions on anticorrosion properties of Ca-doped TiO_2_ bioactive sol-gel coatings. Ceram. Int..

[39] Tsuchiya H., Macak J.M., Müller L., Kunze J., Müller F., Greil P., Virtanen S., Schmuki P. (2006). Hydroxyapatite growth on anodic TiO_2_ nanotubes. J. Biomed. Mater. Res..

